# Malaria prevention measures in Burkina Faso: distribution and households expenditures

**DOI:** 10.1186/s12939-014-0108-0

**Published:** 2014-11-07

**Authors:** Fadima Yaya Bocoum, Danielle Belemsaga, Alex Adjagba, Damian Walker, Seni Kouanda, Halidou Tinto

**Affiliations:** Département biomédical et santé publique 03BP 7192 Institut de Recherche en Science de la Santé, Ouagadougou, Burkina Faso; SIVAC (Supporting National Independent Immunization and Vaccine Advisory Committees) Initiative, Agence de Medecine Preventive (AMP), Paris, France; Integrated delivery, Bill and Melinda Gate Foundation, Seattle, WA USA; Institut Africain de santé publique (IASP), Ouagadougou, Burkina Faso; Institut de Recherche en Sciences de la Sante (IRSS), Direction Régionale de l’Ouest (DRO), Bobo Dioulasso, Burkina Faso; Clinical Research Unit of Nanoro (CRUN), Nanoro, Burkina Faso; Laboratory of Parasitology and Entomology, Centre Muraz, Bobo-Dioulasso, Burkina Faso

**Keywords:** Malaria, Prevention, Expenditures, Households, Burkina faso

## Abstract

**Background:**

The provision of insecticide-treated nets (ITNs) is widely accepted in Burkina Faso thanks to large-scale national distribution campaigns. However, household also use other methods of prevention. Thus far, there is little knowledge about the expenditures of these malaria prevention methods, particularly in combination with the national interventions. This paper presents the utilization levels and expenditures of malaria prevention tools in Burkina Faso and explores the potential inequality in ownership.

**Methods:**

The analysis is based on a cross-sectional survey, conducted during the 2010 high transmission season from July to September in the Nanoro Health and Demographic Surveillance Site. Following a systematic sampling technique, the survey covers 500 households with children under 5 years of age from 24 villages.

In the survey, households were asked about expenditures on malaria prevention methods in the month preceding the survey. This includes expenditure on coils, indoor spraying, aerosols, repellents, herbs, cleaning of the environment and clearing of the vegetation. The data analysis was conducted with SPSS taking into account the socio-economic status (SES) of the household to examine any differences in the utilization of the prevention method and expenditure quintiles. An asset-based index, created through principal components analysis (PCA), was used to categorize the households into quintiles.

**Findings:**

Of the households surveyed, 45% used one preventive measure in the past month; 29% used two measures; and 25% used three or more measures. A significant association was found between the number of prevention measures and the SES of the household (p < 0.05). The majority of households owned at least one insecticide treated net (ITN) (98%). Among households that used ITN, 53.8% used methods other than bed nets. The majority of households paid nothing for malaria prevention.

**Conclusion:**

Most of the households received bed nets and other preventive method for free. There is equity in expenditures across SES groups. Free distribution of ITNs ensured that there was equity in ITN ownership among households. More research on the possibility of increasing access to other locally relevant methods of malaria control that proved to be effective is need.

## Introduction

Malaria is the most preeminent tropical parasitic disease and one of the top three killers among communicable diseases [1]. More than one-third of the world’s population is at risk with the negative consequences of the disease affecting children, adults, and the development of societies. In 2012 malaria caused an estimated 627,000 deaths; most of them were young children in sub-Saharan Africa [2]. In addition to death, malaria impacts fertility, population growth, causes absenteeism in both children and adults, thereby affecting productivity, savings and investment [3–5]. In Burkina Faso, malaria remains a major cause of morbidity and mortality, accounting for over 48% of outpatient visits and about 54% of deaths in hospital patients. Malaria is the leading cause of under-five mortality contributing 85.3% of all childhood deaths [6]. In 2010, the Demographic and Health Survey (DHS) found a prevalence of malaria of 66% in children under the age of 5 [7].

There are a number of powerful and cost-effective methods to prevent malaria. Two of the most common, and most well-studied, are insecticide-treated bed nets (ITNs) and indoor residual spraying (IRS). A series of randomized control trials have demonstrated that insecticide-treated nets are highly effective in preventing malaria in children under 5 years of age [8,9]. The efficacy of IRS in reducing malaria transmission and malaria attacks in children has also been demonstrated [10]; so have dual interventions of ITN-IRS in combination in comparison to single intervention [11–13].

Both ITNs and IRS are national interventions employed in Burkina Faso, where the control of malaria is under the responsibility of the National Malaria Control Program (NMCP). An average coverage of ITNs of about 50% [7] has been achieved in the country, yet their utilization is mitigated by socio-cultural factors [14].

Apart from these interventions, a number of other malaria prevention methods are used in Sub-Saharan Africa and particularly in Burkina Faso, such as repellents, clearing the vegetation around homes, eating or drinking herbs, growing specific plants, and cleaning the home and its surroundings [9]. But little is known about the cost of these malaria prevention methods, particularly in combination with the national interventions [15–17]. This paper presents the level of utilization and expenditures on malaria preventive tools in Burkina Faso and explores potential inequity in ownership.

## Methods

### Study area

This study was conducted in the health district of Nanoro. Nanoro is one of five districts of the Central-West health region of Burkina Faso. The district is located in the centre of Burkina Faso, 85 km west of from Ouagadougou, the capital city. In 2012, the total population was estimated at 149,987 of which 20% were under 5 years old [18]. Within Nanoro, the main road infrastructure is dirt roads and bush paths connecting villages. These roads present a challenge for users especially during the rainy season when there are floods. There are three main ethnic groups (Mossi, Gourounssi and Peulh); most of them are cattle- keepers and/or subsistence farmers. French is the official language, but most of the population speaks the main local language, Mooré. The literacy rate is low; both for men and women (about 23%) [19]. Nanoro district is a Roll Back Malaria (RBM) site for the National Malaria Control Program (NMCP). Malaria is hyper-endemic with seasonal transmission. Malaria is responsible for 56.4% [19] of all health facility consultations throughout the year, with a peak during the rainy season from September to October.

### Study design

A cross-sectional survey was conducted among 500 households with children aged 5 years or younger in the study area. Questionnaires were directed at the head in the household. Respondents were asked about the utilization of different types of malaria preventive tools and any expenditure made in the past month for the prevention of malaria.

### Sampling

Households were selected from 24 villages in the Nanoro Health Demographic Surveillance Site (DSS) [20]. A list of all the households in these villages with children under five years of age who were not enrolled in the ongoing malaria vaccine trial was compiled using the Nanoro DSS house numbering system. From this list every 6th household meeting the inclusion criteria (having at least one child under the age of 5 in the households and not being part of the malaria vaccine trial) was selected, reaching a total sample size of 500 households.

### Data collection

Data collection was conducted from July to September 2010. Interviews with the household respondents were conducted by field workers trained by the researchers, using questionnaires. Having obtained written informed consent, data was collected on the ownership, utilization, and expenditures of bed nets, indoor residual spraying and other malaria preventive methods such as mosquito repellents, smoke, clearing of vegetation, eating/drinking herbs, mosquito coils, cleaning the home and its surroundings, and growing plants. Information was also collected on household assets to determine their socio-economic status. In order to validate the questionnaire, a pre-test was conducted and modifications were made to the questionnaire prior to the beginning of the main data collection. If the household head was not available, the family was asked to nominate the next most appropriate person to respond.

### Data analysis

The data analysis was conducted using SPSS. Differentiating by socio-economic status (SES) we examine differences in the utilization of prevention method utilization and expenditures. Data on asset ownership, water sanitation, type of house, and size of household were collected and used to generate the SES index. The selected assets were: bicycles, motorbikes, radios, television sets, cows, sheep, chicken and cellular phones. The SES is an asset-index, created using principal components analysis (PCA), was used to categorize the households into quintiles: richest (Q5), richer (Q4), poor (Q3), very poor (Q2) and poorest (Q1). Data were analyzed using tabulations, equity analysis, descriptive statistics and nonparametric tests. The equity analysis looked at the distribution of a variable among socio-economic status, i.e. by household (quintile). Nonparametric test like the Kruskall Wallis test was performed to test for association between socio-economic status and prevention expenditures. The currency was in CFA and converted in USD with 1 USD =492 CFA.

### Ethical issues

Ethical clearance was obtained from the National Ethics Committee for Health in Burkina Faso and the Ethics Review Committee of PATH. In addition, the study team obtained permission to conduct the study from the Nanoro District authorities. Written informed consent was obtained from all participants.

## Results

### Socio-demographic characteristics of households

The majority of the household heads were men (92%). The mean age was 48 years. The average size of the households in the sample was 10 persons. On average the households have 2 children below the age of 5 (Table 1).

**Table 1:** **Socio-demographic characteristics of households (N = 500)**

**Variables**	**n (%)**
Gender of head of household	Male: 460 (92)
	Female: 40 (8)
Average age of head of household (year) (SD)	47.7 (15.05)
Average size of household (SD)	10.4 (6.4)
Average number of under 5 years old children (SD)	1.8 (1.2)

### Ownership and use of bed nets

**Figure 1:**
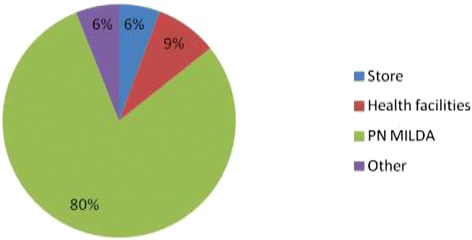
**ITN sources.**

The majority of households owned at least one insecticide treated net (ITN) (98%). The average number of ITNs per household was 5 (±15). The net person ratio was one net for every two persons. Figure 1 shows the sources from which the ITNs have been obtained. Most ITNs (80%) were provided by the national distribution campaign (Programme National Moustiquaires Imprégrnées à Longue Durée d’Action (PN MILDA)), 9% were from public health facilities and 6% were purchased in a store. The average duration of ownership was 5 months (±23 days). ITNs were reported to have been used the previous night by 94% of the heads of the household. 88% of the children under 5 slept under a net in the previous night; so did 85% of the partners and 83% of the children older than 5 years.

### Number of methods of prevention

**Figure 2:**
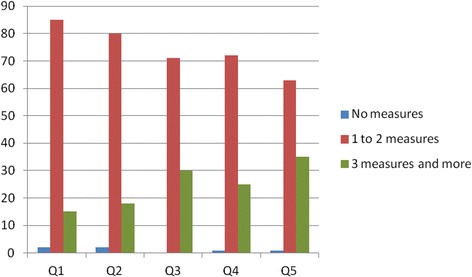
**Number of preventive measures according to socio-economic quintile.**

Of the households surveyed, 45% reported to have used one preventive measure in the past month; 29% used two measures and 25% used three or more measures. Only 1% of the households did not use any preventive method in the previous month. A significant association was found between the number of prevention measures and the SES of the households (p < 0.05). Figure 2 shows the distribution of the number of preventive measures across the socio-economic quintiles. The majority of households in the poorest quintile used 1 to 2 measures and those in the richest quintile were more likely to use 3 or more measures.

### Other preventive methods

Table 2 shows that among households that used any method of prevention, 53.8% used methods other than bed nets. Of these, the method most frequently used was smoke (32.6%), followed by cleaning the home and its surroundings (20.6%). Mosquito repellents and clearing vegetation were the least used.

**Table 2:** **Utilization of other preventive methods (N = 500)**

**Preventive method**	**%**
Mosquito repellents	0.2
Smoke	32.6
Clearing vegetation	2 .6
Eating/drinking herbs	3.2
Mosquito coils	7.8
Cleaning the home and its surroundings	20.6
Indoor residual spraying	3.0
Growing plants	16.0

The richest SES quintile was the only group that used mosquito repellents as preventive tool. There was a significant association between cleaning the home and its environs and the SES quintile (p < 0.05). The richest quintile cleaned the home and its environs more than others. The poorest quintile was more likely to use smoke as preventive method.

### Expenditures of malaria prevention

The majority of households paid nothing for malaria prevention (94% of bed nets and 91% of other preventive methods were provided free of cost). Among households that paid for malaria prevention, the mean expenditure for obtaining a bed net was 2,464 CFA (5 USD) (±1 410 (2.87 USD)). The median expenditure for other preventive methods was 1,500 CFA (3.05 USD) and the mean expenditure was 1,583 CFA (3.21 USD). There was a significant association between the expense on other malaria prevention methods and the SES of the household (p < 0.05). In line with expectations, the poorest households spent less on other prevention tools than the richest ones (Table 3).

**Table 3:** **Cost of other preventive methods by wealth quintile (Kruskal Wallis Test)**

**SES quintile**	**N**	**Mean rank**
Q1	101	237,28
Q 2	100	237,68
Q 3	101	257,13
Q 4	98	250,74
Q 5	99	267,42
Total	499	237,28

P =0.01; Chi-square =13.011; df =4.

## Discussion

This study showed that the majority of households did not pay for prevention measures. Among those who paid, a monthly average of 1,583CFA (3.21 USD) was spent on prevention. This represents approximately 5% of the national minimal monthly salary for agricultural activity. This figure could not be extrapolated across the entire year because expenditures vary by season (wet versus dry). The monthly expenditure reported in this study is less than that in previous studies in Africa (2,216CFA (4.50 USD)) in Burkina Faso [21] and 2,377CFA (4.83 USD) in Cameroon [22]). However, these studies were conducted in urban areas, where prices are likely to be higher. By contrast, our study was conducted in a rural area and the results are likely to be more representative of the majority of the country, as 70% of the population in Burkina Faso resides in rural areas [23]. The poorest households in this study spent less on preventative measures compared to the richest ones suggesting that there is equity in the financial burden of the preventative measures across the socioeconomic groups. Our findings are consistent with other studies that found wealthier households spent more on malaria prevention [24]. In addition to wealth, other determinants such as location of residence and household occupation have been identified as important factors in household expenditure on malaria prevention [25].

The number of prevention measures used was found to vary by socio-economic quintiles. The richest quintile used more prevention measures than the poorest. The most common preventive measures were bed nets. In addition to bed nets, smoke and cleaning the home and its environs are also commonly used, but only cleaning the home and its environs was associated with the household SES. Regarding the number of preventive measures households owned, we could formulate the hypothesis that people use different protective measures because they think that one prevention method is not effective.

Concerning the type of measure, households used measures without proven efficacy (herb, smoke, growing plants, etc.) but which are locally acceptable and affordable. In view of these behaviors, it could be interesting to develop or promote local preventive methods with proven efficacy.

The majority of households owned at least one ITN (98%); the net person ratio was one net for every two persons. The level of ownership is higher than the national average where 56% of the households have at least one ITN [7]. Ownership may have been biased upwards because the national net distribution campaign (PN MILDA) was launched in the Nanoro district in July 2010 and our survey started in the very same month. This finding supports the existing evidence that free distribution ensures higher coverage of ITNs [26,27]. However, strict quality controls are needed, as it was found that approximately one-third of the nets distributed in 2010 in Burkina Faso were not treated with insecticide [28]. In 2010, the DHS survey showed inequity in ITN ownership among households: 63% of the richest households had at least one ITN versus 48% of the poorest ones. However, other studies have found a more equitable distribution of ITNs [29,30]. The difference might be explained by the level of access to the various points of net distribution and stock out that occurred during the campaign.

Despite the fact that we found that the highest rate of reported ITN usage among adult male heads of household (94%), as has been documented in other studies [9], utilization of ITN among vulnerable groups, such as <5 years old, was high in our study (80%) compared to the national average (53%) [7]. Our study could not assess whether nets were being used properly. Toé et al. found that there are barriers to proper utilization of mosquito nets, e.g. due to space within the household [14] and practicality when residents sleep outside due to high temperatures [21]. According to Ahmed et al., a very small proportion (<5%) are knowledgeable about all norms for insecticidal bed net use [27]. Our study could not assess equity in utilization among socio-economic groups, however a 2010 national survey showed inequity in bed net utilization: 26% in the poorest group use bed nets versus 34% respectively 33% in the rich or richest quintile [7].

## Conclusion

In conclusion, the majority of households did not pay for malaria prevention methods due to the free distribution of bed nets and use of traditional methods. The findings suggest that there is equity in ITN ownership and expenditures among socio-economic groups in rural Burkina Faso. The use of traditional methods calls for more research on the possibility of increasing access to locally relevant methods of malaria control that have proven efficient.

## Abbreviation

PN MILDA: Programme National Moustiquaires Imprégrnées à Longue Durée d’Action
